# First clinical experience with IVR-CT system in the emergency room: Positive impact on trauma workflow

**DOI:** 10.1186/1757-7241-20-52

**Published:** 2012-08-07

**Authors:** Daiki Wada, Yasushi Nakamori, Kazuma Yamakawa, Satoshi Fujimi

**Affiliations:** 1 Department of Emergency and Critical Care, Osaka General Medical Center, 3-1-56 Bandai-Higashi, Sumiyoshi-ku, Osaka, 558-8558, Japan; 2 Department of Traumatology and Acute Critical Medicine, Osaka University Graduate School of Medicine, 2-15 Yamadaoka Suita, Osaka, 565-0871, Japan

## Abstract

Recently, computed tomography (CT) has gained importance in the early diagnostic phase of trauma care in the emergency room. We implemented a new trauma workflow concept with CT in our emergency room that allows emergency therapeutic intervention without relocating the patient. Times from patient arrival to CT initiation, CT end, and definitive intervention were significantly shorter with our new protocol than were those with the conventional CT protocol. Our new workflow concept, which provides faster time to definitive intervention, appears to be effective.

## Findings

### Background

Recently, computed tomography (CT) in the emergency room has become an essential part of trauma diagnostic workups. Hilbert et al. reported that eliminating patient transfer from the emergency room to the CT scanner location is of enormous benefit [1]. We implemented a new trauma workflow concept with a sliding CT scanner system with interventional radiology features (IVR-CT). The purpose of this study was to evaluate the therapeutic value of this workflow concept in terms of workup times.

### Materials and methods

#### Patient population

This historical control study was conducted from February 2010 to April 2012 in a level I trauma center in Japan. Inclusion criteria were patients with blunt trauma who were admitted directly from the incident scene and required emergency bleeding control, which was defined as any emergent thoracotomy, laparotomy, or transcatheter arterial embolization (TAE). Patients in traumatic cardiorespiratory arrest at arrival were excluded. The institutional review board of Osaka General Medical Center approved the conduction of this study.

**Figure 1:**
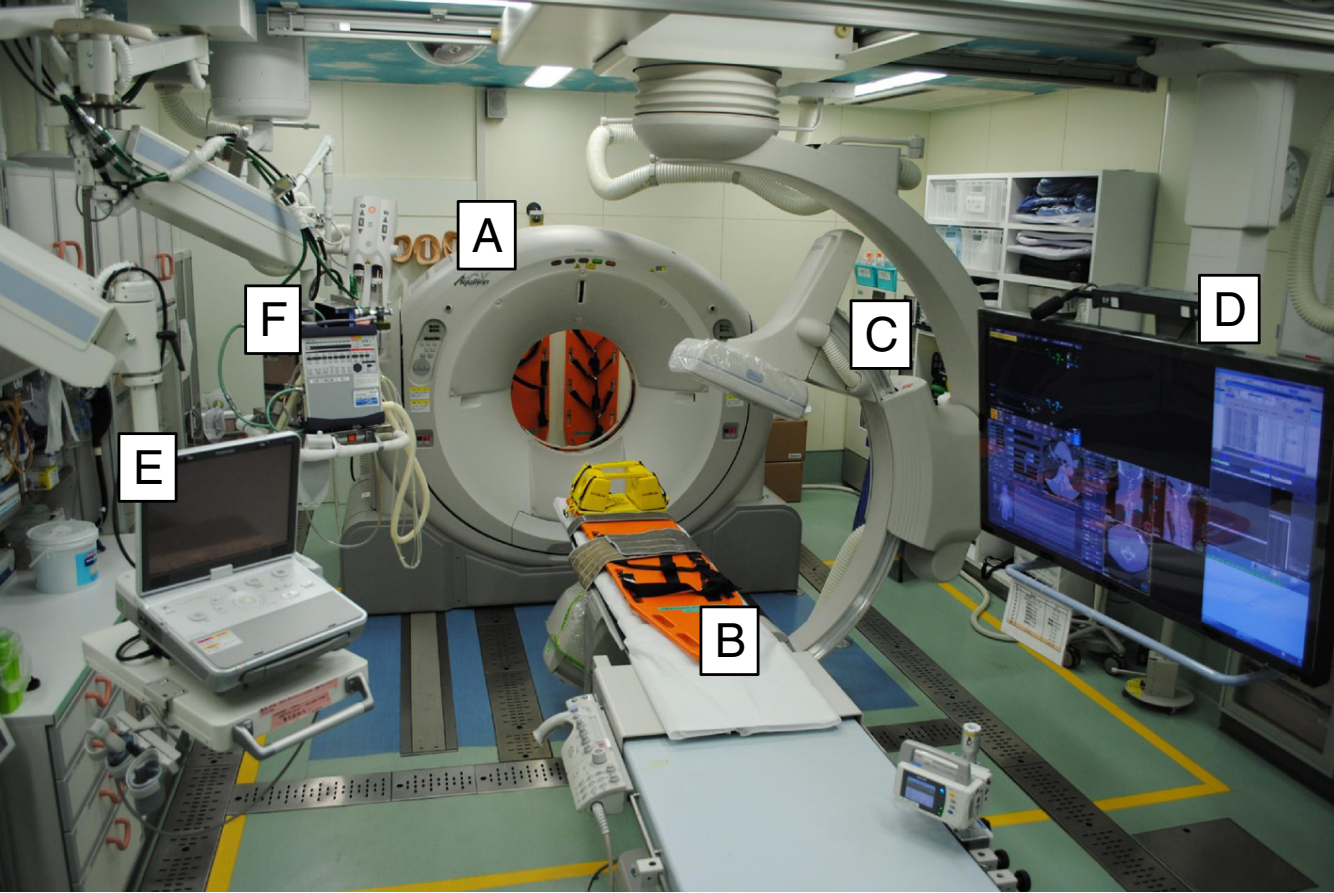
**Photograph showing the IVR-CT system in our emergency room.** All life-saving procedures including airway management, emergency surgery, and TAE can be performed on the table without relocating the patient. (**A**) sliding CT scanner, (**B**) CT examination and intervention table, (**C**) moveable C-arm, (**D**) 56-inch monitoring screen, (**E**) ultrasonography equipment, and (**F**) mechanical ventilator.

#### Setting

Before the introduction of IVR-CT in our trauma workflow concept, the diagnostic measures used in the early treatment phase of trauma care included conventional radiography in combination with focused assessment with sonography for trauma. After completion of radiography and sonography, patients were transferred to the CT scanner, which was located on the same floor as the emergency room.

In August 2011, a new multislice IVR-CT system (Aquilion CX, TSX-101A; Toshiba Medical Systems Corp., Tochigi, Japan) was installed in our emergency room. All life-saving procedures including airway management, damage control surgery, and TAE can be performed on the CT examination and intervention table (Figure 1).

#### Data collection

Main outcome measures were time from patient arrival to CT initiation, CT end, and to the start of emergency bleeding control procedures. To evaluate the effects of this new workflow concept, data from two groups of patients were analyzed. The IVR-CT protocol group included patients admitted between August 2011 and April 2012. The conventional CT protocol group included patients admitted between February 2010 and July 2011. We also assessed 28-day mortality.

#### Statistical analysis

Data are expressed as group medians with interquartile ranges or numbers with percentages, as appropriate. Continuous variables were compared between groups with the Mann–Whitney *U* test. Categorical variables were analyzed with the *χ*^2^ test or Fisher’s exact test, as appropriate. A *P* value of <0.05 was considered statistically significant.

### Results

#### Baseline characteristics

During the study period, 48 consecutive patients fulfilled the inclusion criteria. Twenty-one patients were eligible for the IVR-CT protocol and 27 patients for the conventional CT protocol. No significant differences in baseline patient characteristics were found (Table 1). One patient in the IVR-CT protocol group and 7 patients in the conventional CT protocol group were transferred to the operating room for emergency surgery.

**Table 1:** Baseline Patient Characteristics

**Characteristic**	**IVR-CT protocol (n = 21)**	**Conventional CT protocol (n = 27)**	***P*-value**
Patient characteristics			
Age, yrs	44 (35–56)	48 (33–57)	0.795
Male sex	15 (71)	18 (67)	0.764
Severity of trauma			
SBP, mmHg	115 (102–134)	122 (97–140)	0.875
Heart rate, bpm	105 (80–125)	104 (82–130)	0.976
RTS	7.8 (6.0-7.8)	7.6 (6.9-7.8)	0.664
Hemoglobin, g/dl	12.4 (11.4-14)	12.4 (11.2-13.4)	0.260
Prothrombin time, %	54 (44.7-71)	51.7 (46.7-71.6)	0.547
ISS	38 (26–44)	34 (26–43)	0.573
TRISS	13.1 (3.7-57.2)	12.2 (4.9-21.3)	0.662
Therapeutic interventions			
Blood transfusion within 24 hrs, unit	10 (2–16)	14 (8–24)	0.335
Bleeding control site			
Chest	6 (29)	2 (7)	0.115
Abdomen	9 (43)	14 (52)	0.573
Pelvic	14 (67)	12 (44)	0.153
Bleeding control procedure			
TAE	14 (67)	2 (10)	1.000
Thoracotomy	5 (24)	18 (67)	0.574
Laparotomy	1 (4)	8 (30)	0.750

Data are expressed as group medians (interquartile ranges) or number (percent).SBP, systolic blood pressure; Heart rate per a minute; bpm, beat per minute; IVR, interventional radiology; CT, computed tomography; RTS, Revised Trauma Score; ISS, Injury Severity Score; TRISS, Trauma and Injury Severity Score; TAE, transcatheter arterial embolization.

#### Diagnostic and therapeutic procedures

Time results are shown in Table 2. Compared with the conventional CT protocol, all workup times including time to the start of bleeding control procedures were significantly shorter with the IVR-CT protocol. There was no significant difference in 28-day mortality between the two groups.

**Table 2:** Outcomes Related to Time Analysis

	**IVR-CT protocol (n = 21)**	**Conventional CT protocol (n = 27)**	***P*-value**
Time to CT initiation, min	10 (5–17)	29 (25–39)	<0.001
Time to CT end, min	16 (12–23)	38 (33–48)	<0.001
Time to definitive therapy, min			
Thoracotomy/laparotomy^a^	45 (37–65)	108 (81–129)	0.004
Transcatheter arterial embolization^b^	54 (42–66)	75 (58–105)	0.007
28-day mortality, %	5 (24)	5 (19)	0.729

Data are expressed as group medians (interquartile ranges) or number (percent).^a^ Comparison between IVR-CT protocol (n = 7) and conventional CT protocol (n = 9).^b^ Comparison between IVR-CT protocol (n = 14) and conventional CT protocol (n = 18).

IVR, interventional radiology; CT, computed tomography.

### Discussion

In recent years, many major urban trauma centers have elected to install CT scanners close to or inside their emergency rooms. Although this concept substantially diminishes delays resulting from transferring patients to CT scanners, issues of patient transfer to specialized departments for definitive therapy remain as one of the rate-limiting steps in achieving maximum patient throughput. The primary advantage of our IVR-CT protocol over those of previous reports is the ability to start emergency bleeding control procedures without transferring the patient to the radiology department or operating room after completion of the diagnostic workup. Several studies previously reported that times to the start of emergency bleeding control procedures were about 80–100 minutes [2,3], whereas those times in our trauma protocol were 45 minutes for surgery and 54 minutes for TAE, obviously shorter than those of these previous reports. However, a possible disadvantage of this workflow concept is that the emergency room can be occupied for a long period of time during bleeding control procedures.

Clarke et al. reported that delay to laparotomy in patients with intra-abdominal hemorrhage after trauma was associated with an increased risk of mortality [4]. Time to complete diagnostic tests and initiate definitive therapy, the “golden hour”, is frequently mentioned in the care of seriously injured patients. Weninger et al. reported that a new management algorithm with sliding CT leads to a reduction in organ failure rates [3]. In addition, Wurmb et al. reported that the rapid diagnostic workup with sliding CT might be associated with an improved outcome [5]. Thus, our IVR-CT-based workflow concept allowing faster definitive intervention may have an impact on patient survival.

We acknowledge several limitations in this study. First, this is a retrospective historical control study and not a randomized control study. Second, this study was conducted in a single institution. Third, the sample size was small. Thus, further study is required to evaluate the value of this workflow concept, including adverse events such as wound infection or sepsis that could be related to the performance of surgical procedures in the emergency room.

### Conclusion

Our new trauma workflow concept using an IVR-CT system in the emergency room can facilitate definitive interventions more quickly in comparison with a conventional CT protocol.

## Abbreviations

CT: computed tomography; IVR: interventional radiology; TAE: transcatheter arterial embolization.

## Authors' contributions

DW participated in study design and in data collection and interpretation and drafted the manuscript. YN conceived the study and its design and helped to draft the manuscript. KY had a major impact on the interpretation of data and critical appraisal of the manuscript. SF participated in data interpretation. All authors read and approved the final manuscript.

## Competing interests

The authors declare that they have no competing interests.
